# Structural Recognition and Binding Pattern Analysis of Human Topoisomerase II Alpha with Steroidal Drugs: *In Silico* Study to Switchover the Cancer Treatment

**DOI:** 10.31557/APJCP.2020.21.5.1349

**Published:** 2020-05

**Authors:** Qazi Mohammad Sajid Jamal

**Affiliations:** *Department of Health Informatics, College of Public Health and Health Informatics, Qassim University, Saudi Arabia. *

**Keywords:** Topoisomerase, steroidal drugs, anticancer, docking, binding energy

## Abstract

**Background and objective:**

Topoisomerase TOP-IIA (TTOP-IIA) is widely used as a significant target for cancer therapeutics because of its involvement in cell proliferation. Steroidal drugs have been suggested for breast cancer treatment as aromatase enzymes inhibitors . TTOP-IIA inhibitors can be used as a target for the development of new cancer therapeutics.

**Materials and Methods:**

In this study, we conducted a docking study on steroidal drugs Anastrozole (ANA), Letrozole (LET), and exemestane (EXE) with TTOP-IIA to explore the therapeutic area of these drugs.

**Results:**

The binding interaction of EXE drug had significant docking interaction which is followed by ANA and LET. Thus, all these drugs could be used to inhibit the TTOP-IIA mediated cell proliferation and could be a hope to treat the other types of cancers. Among all three tested steroidal drugs, EXE showed binding energy -7.05 kcal/mol, hydrogen bond length1.78289 Å and amino acid involved in an interaction was A: LYS723:HZ3 -: UNK1:O6.

**Conclusion:**

The obtained data showed the most significant binding interaction analyzed with the tested enzyme. Thus, *in vitro* laboratory experimentation and* in vivo *research are necessary to put forward therapeutic repositioning of these drugs to establish them as a broad spectrum potential anticancer drugs.

## Introduction

The topology of DNA is maintained by nuclear enzyme topoisomerases. They catalyze the provisional cutting and ligation processes of DNA, and classified as topoisomerases TOP-I (TTOP-I), TTOP-IIA, and topoisomerase TOP-IIB (TTOP-IIB) (Gupta et al., 1995). The TTOP-I cuts single-stranded DNA without the expenditure of ATP (Lin et al., 2014), while topoisomerase-II cuts DNA from double-strand with the use of ATP. TTOP-IIA catalyzes a cut on the 3′ end of DNA, while TTOP-IIB catalyzes by covalently linked to the 5’ end of the DNA (Pommier et al., 1994). The topoisomerase IA is associated with dividing the nature of the cells, while Topo B is found in neuron and not associated with dividing the nature of the cells. According to previously published research, TTOP-I is associated with cancer, and TTOP-II plays a significant role in cancer development. Thus, these enzymes contribute much more for cancer detection and treatment (Pommier et al., 1998; Andoh and Ishida 1998; Nitiss 2009). 

TTOP-IIA is widely used as a significant cancer therapeutic because of its involvement with cell proliferation. In many cancer cells, the divergent expression of TTOP-IIA has been reported. Due to this characteristic, it can be utilized as a biomarker for the diagnosis of different types of cancer (Pommier et al., 1994; Yousaf et al., 2015). This study presented the topoisomerase integration and analysis to explore the understanding of TTOP-IIA in various carcinomas. Therefore, many scientific reports show contradictions within a similar area of study, the outline of this study that may help to accelerate the research for additional exploration of cancer therapeutics . A previous topoisomerase inhibitor analysis depicted the Aromatase inhibitor (AI) as a sweet overtreatment for various types of carcinomas particularly in liver carcinoma (Bromberg and Osheroff, 2003; Wang, 1996; Thakur, 2011). Wang (1996) a biochemist from America, for the first time revealed that topology of *Escherichia coli* DNA was regulated by a special nuclear enzyme. The telomerase activity was also reported in other prokaryotes and eukaryotes including, humans (Lin et al., 2014). Many compounds that work as poisons for topoisomerase work as anticancer drugs that cleave complex of DNA and enzyme. Topoisomerase can be linked to breast cancer and its overexpression has been seen in many cancers. Thus, analysis of implication, overexpression, and co-amplification of many genes can contribute to understanding of the stages of cancer. Under urinary bladder cancer, ovarian carcinoma, prostate cancer, soft tissue sarcoma, brain-related cancers, non-small cell lung cancer and hepatic cancers (Bromberg and Osheroff, 2003). 

AIs block estrogen synthesis and thus have been tested well as an alternative treatment for estrogen-dependent breast cancer. They act through inhibiting the function of the aromatase enzyme complex, which catalyzes this conversion (Brodie et al., 1977) and incapable of binding on its actual site of androgen substrate. Aromatase forms estrogen in ovaries and in several tissues of the body (Reva et al., 2011). AIs are divided into type I, steroidal and type II, non-steroidal. The steroidal ones are formestane and EXE which are structurally similar to natural substrates, testosterone ,and androstenedione (Miller et al., 2008). 

They are more beneficial and safe in clinical studies than tamoxifen. Aromatase is a cytochrome P450 enzyme complex that expresses *CYP19* gene at chromosome number 15q21.2. Its expression is found in ovaries, extragonadal tissues, fat, brain, liver, bones, endothelium, and mesenchymal cells of adipose tissue in the breast. Aromatase converts androgens (androstenedione and testosterone) to estrogen and estradiol (Zhao et al., 2016). 

First, aromatase inhibitor, formestane, was used to compare it with tamoxifen effect in clinical trials and similar effect was reported. Other AIs, including ANA (Bonneterre et al.,2000), LET (Mouridsen et al., 2003), and EXE (Mokbel et al., 2002; Paridaens et al., 2003; Rocío et al., 2013) had higher efficiency when used in combination compared to use tamoxifen alone. AIs ANA and LET are given as a first therapy to post-menopausal hormone-sensitive women having advanced breast cancer (Mouridsen et al., 2003; Cuzick et al., 2010). However, AIs are not given to premenopausal women since they are not able to inhibit the enzyme as it is formed more in ovaries of these women (Mauri et al., 2006). These drugs are effective in these women when given in combination with other drugs inhibiting ovarian function (National Cancer Institute, 2012). Few side effects are reported for AIs, such as blood clots, stroke, bone loss, and heart problems (Breast Cancer Organization, 2014). Aromatase inhibitor is used for the prevention and treatment of breast cancer in patients whose ovaries stop functioning. Inhibitors have been used to activate ovulation in anovulatory infertility. They are used in combination with tamoxifen (Usluogullari et al., 2015). AIs reduce the recurrence and side effects, hence increasing survival of breast cancer patients. AIs inhibit estrogen synthesis by binding to the estrogen receptors and modulate cell proliferation. Topoisomerases are also involved in cell proliferation, AIs can be considered as an alternative treatment to inhibit topoisomerases activity. Computational methods like molecular docking analysis help us to identify the interaction and binding pattern of ligand molecules with receptor biomolecules and using 3 Dimensional (3D) visualization techniques it could be well depicted in the form of graphics for better understanding. Through implementing docking techniques, many studies have been already done to find out better TTOP-IIA inhibitors. Drwal et al., (2014) tested TTOP-IIA inhibitors through computational virtual screening methods and identified 8 potential compounds. Another docking study suggested camptothecin as TTOP-IIA inhibitor (Laco et al., 2002). AIs are well trusted against breast cancer. To explore the therapeutic application of AIs and their interaction, *in silico* studies are conducted using topoisomerase. Therefore, in this study, TTOP-IIA inhibitions were tested with steroidal drugs. 

## Materials and Methods

*Drug molecule preparation*

**Figure 1:**
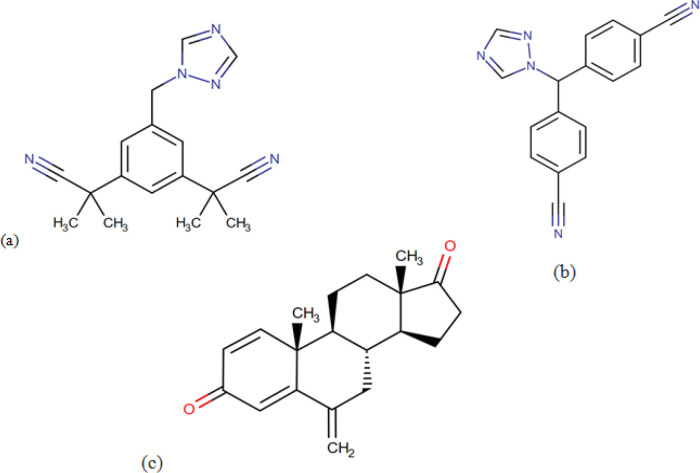
2D Structures of Drug Compounds (a), ANA; (b), EXE; (c) LET extracted from the DrugBank database

Two-dimensional (2D) structures of drugs molecule ANA (DB01217) (Figure 1(a) ), EXE (DB00990) (Figure 1(b)) and LET (DB01006) (Figure 1 (c)) (Table 1) were downloaded from DrugBank Database (www.drugbank.ca) (Wishart et al., 2018). The 2D drugs compound structure cannot be used directly to run docking studies and it should be further converted to .pdb files and minimized using CHARMm forcefield (Brooks et al., 2009) energy minimization procedure available in Discovery Studio visualizer 2019v (Dassault Systèmes BIOVIA, 2019).

*Receptor molecule preparation*

**Figure 2:**
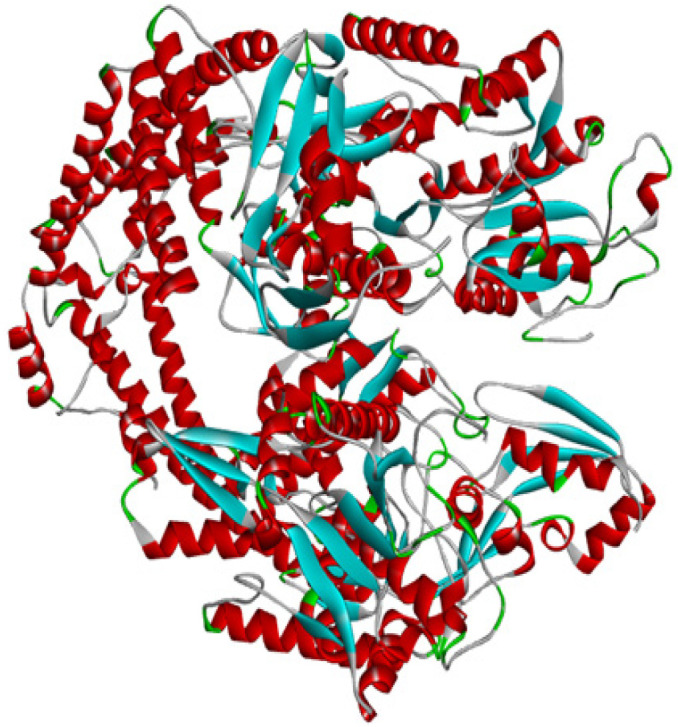
3D Crystallography Structure of HTIIa (PDB ID: 5GWK)

The well-known protein structure database Protein Data Bank (PDB) governed by Research Collaboratory of Structural Bioinformatics (RCSB) (www.rcsb.org) (Berman et al., 2000) was used to download the 3D structure of human topoisomerase II alpha (HTIIa) (PDB ID: 5GWK) (Figure 2). The structure was selected on the basis of available experimental data like the X-ray diffraction method used to develop the 3D structure with the obtained resolution of 3.152 Å, R-value Free 0.244, and R-value work 0.203 (Wang et al., 2017). The water molecules and HETATM were removed from the 5GWK.pdb file, and CHARMm force field was applied for energy minimization (Brooks et al., 2009) using Discovery Studio visualizer 2019v (Dassault Systèmes BIOVIA, 2019).

*In silico interaction analysis*

MGL tool Autodock was used to predict the binding affinity between drug compounds and topoisomerase enzymes. Interaction analysis was carried out using the Lamarckian genetic algorithm (LGA). Molecular docking methods followed by probing the best conformation of enzyme and drugs compound-complex on the calculation and obtained binding energy (∆G). 

∆G binding = ∆Ggauss + ∆Grepulsion + ∆Ghbond + ∆Ghydrophobic + ∆Gtors 

In the above mentioned formula, ∆G gauss is dispersion of two gaussian functions, ∆Grepulsion is square of the distance if closer than a threshold value, ∆Ghbond is ramp function, it is also used for interactions with metal ions, ∆Ghydrophobic is ramp function, and ∆Gtors is proportional to the number of rotatable bonds (Morris et al., 1998; Morris et al., 2009). 

Further, water molecules were removed from the 3D structure of enzyme (i.e. PDB ID: 5GWK). Before docking and hydrogen atoms, Kollman united charges and salvation parameters were added to the 5GWK. Gasteiger charge was added to the drug compounds. The Grid box was set to cover the maximum part of 5GWK and drug compounds. The values were set to 60×60×60° in X, Y, and Z-axis of a grid point. The default grid points, spacing was 0.375 Å. Lamarckian Genetic Algorithm (LGA) (Goodshell et al., 1996 and Tsai et al., 2012) was used for 5GWK-drug compounds flexible docking calculations. The default LGA parameters like population size (ga_pop_size), energy evaluations (ga_num_generation), mutation rate, crossover rate, and step size were set to 150, 2500,000, 27,000, 0.02, 0.8 and 0.2 Å, respectively. The LGA runs were set at 10 runs. After successful execution of docking steps, obtained conformations of 5GWK and drugs-compound complexes were analyzed for the interactions and binding energy using Discovery Studio 2019v molecular visualization software (Dassault Systèmes BIOVIA, 2019).

## Results

The topological properties of DNA are regulated by enzyme topoisomerase (Wang et al., 2002). It also has been involved in DNA repair, transcription, recombination, and replication (Marinello et al., 2013). One of the major ways to kill cancer cells through anticancer drugs is inhibition of TTOP-II (Azarova et al., 2007). Thus, topoisomerase is the target of many drugs, including fluroquinones, mitoxantrone, etoposide, doxorubicin, and amascrine. Its ligand-protein interaction has been analyzed by *in silico* docking studies (Shen et al., 1989). Previous computational analysis revealed that fluoroquinolines could target TTOP-IIA inhibitors during cancer treatment (Jadhav et al., 2017). A recent study used molecular docking techniques and identified rubidazone and other ligands, such as daunorubicin, m-amsacrine, bisantrene, and mitoxantrone, as the best TTOP-II inhibitors (Arthur, 2019). Thus, steroidal drugs could be considered as an alternative drugs to inhibit the targeted enzymes.

Therefore, in this study, we analyzed the binding interaction affinities of ANA, EXE, and LET with topoisomerase II by implementing *in silico* docking studies. All three tested steroidal drugs had significant binding affinities with HTIIa in this study. These drugs have already been used as breast cancer drugs. In this study, an interaction analysis was performed to explore the therapeutic area of these drugs given that HTIIa may involve in maintaining the topology of DNA and any default in this mechanism may result in a different cancer. All drugs used in this study significantly interacted with enzyme, which may loss of interacting native structure and result in inhibition of the topoisomerase activity. 

**Figure 3:**
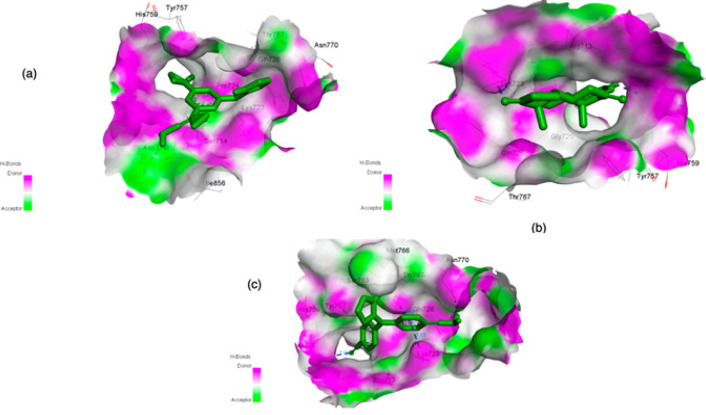
3D Visualization of the Drugs Molecule (a) ANA (b) EXE and (c) LET settlement in the binding pocket of HTIIa (PDB ID:3GWK). Blue dotted lines shows hydrogen bonds. Graphics generated by Discovery Studio Visualizer 2019v

**Figure 4:**
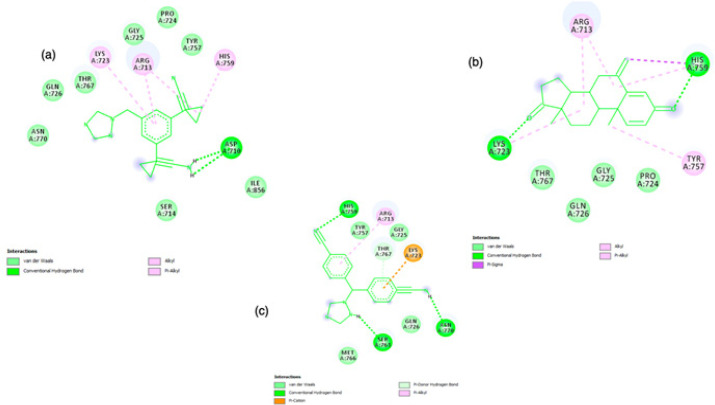
2D Graphics of Drugs Compound (a) ANA (b) EXE and (c) LET binding interactions with HTIIa (PDB ID: 3GWK) and residues involved in the hydrophobic interaction. Graphics generated by Discovery Studio Visualizer 2019v

The docking interaction of ANA with HTIIa showed -6.04 kcal/mol binding energy. The negative value of interacting binding energy indicated significance of binding interaction. The analyzed inhibition constant was 277.73 uM, signifing the binding interaction between ANA and HTIIa. The involved hydrogen bond-forming amino acids were UNK0:H16 - A: ASP710:OD2 and UNK0:H17 - A: ASP710:OD2 with a hydrogen bond length 2.33347 Å and 1.99519 Å, respectively (Figure 3 (a), Table 1). Beside hydrogen bond formation, the hydrophobic interactions were observed with the involvement of amino acid Asp710, Arg713, Ser714, Lys723, Pro724, Gly725, Gln726, Tyr757, His759, Thr767, Asn770, and Ile856. Different types of interaction were also found to provide more stability to the complex structure (e.g. Lys723, Arg713 and His759 involved in alkyl and pi-alkyl interaction) (Figure 4 (a), Table 1).

In addition, analyzing docking interaction of EXE with HTIIa showed -7.05 kcal/mol binding energy. The negative value of interacting binding energy indicated the significance of binding interaction. The analyzed inhibition constant was 6.76 uM, demonstrating the binding interaction between EXE and HTIIa. The involved hydrogen bond-forming amino acids were A: LYS723:HZ3 -: UNK1:O6 and A: HIS759:HE2 -: UNK1:O21 with a hydrogen bond length 1.78289 Å and 2.26904 Å, respectively (Figure 3 (b), Table 1). Beside the hydrogen bond formation, the hydrophobic interactions were also observed with the involvement of amino acid Arg713, Lys723, Pro724, Gly725, Gln726, Tyr757, His759, and Thr767. It was also observed that different types of interaction provided more stability to the complex structure (e.g. Arg713 and Tyr757 involved in alkyl and pi-alkyl interaction and one pi-Sigma interaction found with His759) (Figure 4 (b), Table 1).

Further, the docking interaction of LET with HTIIa showed -6.22 kcal/mol binding energy. The negative value of interacting binding energy of this studied drug also indicated the significance of the binding interaction between LET and HTIIa. The analyzed inhibition constant was 140.71 uM, revealing the binding interaction between ANA and topoisomerase, but this value was less as compared to that of ANA. The involved hydrogen bond-forming amino acids were A: HIS759: HE2 - : UNK0:N3 with a hydrogen bond length 2.96051 Å,: UNK0:H1 - A:SER763:O. Hydrogen bond length of this group was 2.04926 Å,: UNK0:H3 - A: ASN770: OD1 with a hydrogen bond length 2.2549 Å. The other amino acid involved in hydrogen bonding were A: LYS723: HZ3 -: UNK0 and A: THR767: HG1- :UNK0 and with the bond length of 3.12925 Å and 2.59146 Å, respectively (Figure 3 (c), Table 1). In addition to hydrogen bond formation, the hydrophobic interactions were observed with the involvement of amino acid Arg713, Lys723, Gly725, Gln726, Tyr757, His759, Ser763, Met766, Thr767, and Asn770. Same to other interaction analyses in the complex also different interacting bonds found e.g. Lys723 formed pication bond, Arg713 formed pi-alkyl bond to provide more stability to ligand-receptor complex (Figure 4 (b), Table 1). Moreover, it was found that the binding energy of EXE with HTIIa was minimum among the studied drugs. This ANA could be bound more efficiently to inhibit the enzyme more efficiently. 

**Table 1:** Showing Detailed Information of Selected Drugs Molecule

S.No.	Drugs Compound	IUPAC Name	Chemical Formula	Molecular Weight	SMILES ID
1	Anastrozole	2-[3-(1-cyano-1-methylethyl)-5-[(1H-1,2,4-triazol-1-yl)methyl]phenyl]-2-methylpropanenitrile	C_17_H_19_N_5_	293.4 g/mol	CC(C)(C#N)C1=CC(=CC(=C1)CN2C=NC=N2)C(C)(C)C#N
2	Letrozole	4-[(4-cyanophenyl)(1H-1,2,4-triazol-1-yl)methyl]benzonitrile	C_17_H_11_N_5_	285.3 g/mol	C1=CC(=CC=C1C#N)C(C2=CC=C(C=C2)C#N)N3C=NC=N3
3	Exemestane	(1S,2R,10R,11S,15S)-2,15-dimethyl-8-methylidenetetracyclo[8.7.0.0²,⁷.0¹¹,¹⁵]heptadeca-3,6-diene-5,14-dione	C_20_H_24_O_2_	296.4 g/mol	CC12CCC3C(C1CCC2=O)CC(=C)C4=CC(=O)C=CC34C

**Table 2:** Data Obtained through Molecular Interaction Analysis between Drugs Compound and PDB ID:5GWK Receptor Using Auto Dock Tool. The table reflects that EXE has the lowest binding energy -7.05 kcal/mol with 6.76 uM inhibition constant

S.No.	Drugs Compound	Receptor Molecule PDB ID:5GWK	Hydrogen bonds	Length of Hydrogen bond ( Å )	Final Intermolecular Energy (vdW + Hbond + desolv Energy)	Inhibition Constant	Residues involved in hydrophobic interaction
1	ANA	Human topoisomerase II alpha	:UNK0:H16 - A:ASP710:OD2	2.33347	-6.04 kcal/mol	277.73 uM	Asp710,Arg713,Ser714,Lys723,Pro724,Gly725,Gln726,Tyr757,His759,Thr767,Asn770,Ile856
			:UNK0:H17 - A:ASP710:OD2	1.99519			
2	EXE	Human topoisomerase II alpha	A:LYS723:HZ3 - :UNK1:O6	1.78289	-7.05 kcal/mol	6.76 uM	Arg713,Lys723,Pro724,Gly725,Gln726,Tyr757,His759,Thr767
			A:HIS759:HE2 - :UNK1:O21	2.26904			
3	LET	Human topoisomerase II alpha	A:HIS759:HE2 - :UNK0:N3	2.96051	-6.22 kcal/mol	140.71 uM	Arg713,Lys723,Gly725,Gln726,Tyr757,His759,Ser763,Met766,Thr767,Asn770
			:UNK0:H1 - A:SER763:O	2.04926			
			:UNK0:H3 - A:ASN770:OD1	2.2549			
			A:LYS723:HZ3 - :UNK0	3.12925			
			A:THR767:HG1 - :UNK0	2.59146			

## Discussion

This analysis explored the promising potential anticancer activity of AI, and it was observed it could be used as an effective drug against many cancers. Thus, *in vitro *laboratory experimentation and* in vivo* research are necessary to put forward therapeutic repositioning of AI to establish it as a potential anticancer drug against cancers other than breast cancer.

In conclusion, topoisomerases catalyze an important cellular process to maintain the topology of DNA. Their role has also been described in cell growth and development which can be abnormal and resultes in the development of cancers. Many inhibitors have been developed to control cancers originated from the malfunctioning of topoisomerase, including breast, which could be due to the estrogen synthesis . Thus, aromatase inhibitors block estrogen synthesis and can be considred as alternative treatment for estrogen-dependent breast cancer. The *in silico* interaction studies on steroidal drugs signified their therapeutic potentialities against topoisomerases. Among all three tested steroidal drugs, EXE has shown binding energy -7.05 kcal/mol, hydrogen bond length 1.78289 Å and amino acid involved in an interaction was A: LYS723:HZ3 -: UNK1:O6. It was the most significant binding interaction analyzed by a tested enzyme. Thus, in conclusion, *in vitro* laboratory experimentation and *in vivo* research are needed to put forward therapeutic repositioning of these drugs to establish them as a broad spectrum anticancer drugs. 
